# Companies and Medical Practice

**Published:** 1898-06-11

**Authors:** 


### COMPANIES AND MEDICAL PRACTICE.

A matter of very great importance was considered by the General Medical Council at its last meeting. It appears that at the present time it is possible for companies, limited or otherwise, to carry on medical, surgical, and dental practices, and a committee was appointed to take steps to induce the Government to insert a clause in the Companies Act Amendment Bill, now before Parliament, with the object of preventing the registration of companies to carry on such practices. It is a great scandal that, while a medical man is not allowed to employ an unqualified assistant to do his rough work, absolutely unqualified men may be employed by absolutely unqualified companies to practise every branch of medicine. To show how real is the danger to the public which arises from the sanction to illegal practice given by the Companies Act, we would mention that some time ago the Pharmaceutical Society of Ireland forwarded us a memorandum showing how frequently of late companies have been formed apparently for the express purpose of evading the definite provisions of the Pharmacy Acts. The following instances are sufficient to illustrate this point:—

"A leather-dealer, on being warned by the police against compounding medical prescriptions and selling poisons, formed a limited liability company, with a capital of fifty pounds (£50). The shop is styled 'The Medical Hall.'

"A 'chemist and druggist,' after prosecution for compounding, formed a limited company, which carries on the compounding of medical prescriptions.

"An unqualified trader, styling himself a 'druggist,' being warned, formed a limited company, and assumed the title 'chemists and druggists.'

"A firm of medical booksellers and surgical instrument dealers, having, after repeated warnings, been fined for selling poisons, formed a limited company, and now also compound medical prescriptions.

"A retired chandler carried on a 'medical hall.' On inquiries from the police and the society, he formed a limited company.

"An oil and colour dealer, having been fined for selling poison, formed a limited company," &c., &c.

Now, in regard to some of these companies it is to be noted that the things that they were formed to do would, but for the sanction of the Companies Act, have been illegal—far more illegal than the practice of medicine by unqualified persons. We need not, then, be surprised to find that "not only are limited companies registered to transact the business of pharmaceutical chemists and chemists and druggists, but also as apothecaries, and in one case, if not more, to 'carry on the business of physicians and surgeon.'" It would seem, indeed, that whether or not a company is promoted for a "lawful purpose" is not investigated by the Registrar of Joint Stock Companies, and, under these circumstances, there can be no doubt that the modification of law sought by the General Medical Council ought to receive general support.

